# Oxidative Stress and Mitochondria Are Involved in Anaphylaxis and Mast Cell Degranulation: A Systematic Review

**DOI:** 10.3390/antiox13080920

**Published:** 2024-07-29

**Authors:** Anays Piotin, Walid Oulehri, Anne-Laure Charles, Charles Tacquard, Olivier Collange, Paul-Michel Mertes, Bernard Geny

**Affiliations:** 1 Physiology and Functional Exploration Service, Strasbourg University Hospital, 67000 Strasbourg, France; anays.piotin@chru-strasbourg.fr; 2 Division of Asthma and Allergy, Chest Diseases Department, Strasbourg University Hospital, 67000 Strasbourg, France; 3 Team 3072 “Mitochondria, Oxidative Stress and Muscle Protection”, Translational Medicine Federation of Strasbourg (FMTS), Faculty of Medicine, University of Strasbourg, 67000 Strasbourg, France; walid.oulehri@chru-strasbourg.fr (W.O.); anne.laure.charles@unistra.fr (A.-L.C.); olivier.collange@chru-strasbourg.fr (O.C.); paul.michel.mertes@chru-strasbourg.fr (P.-M.M.); 4 Department of Anesthesia and Intensive Care, Strasbourg University Hospital, 67000 Strasbourg, France; charlesambroise.tacquard@chru-strasbourg.fr; 5 Établissement Français du Sang (EFS) Grand Est, French National Institute of Health and Medical Research), (INSERM) BPPS UMR_S1255, Fédération de Médecine Translationnelle de Strasbourg (FMTS), University of Strasbourg, 67000 Strasbourg, France

**Keywords:** oxidative stress, reactive oxygen species (ROS), antioxidant, mitochondria, anaphylaxis, anaphylactic shock, mast cells degranulation

## Abstract

Anaphylaxis, an allergic reaction caused by the massive release of active mediators, can lead to anaphylactic shock (AS), the most severe and potentially life-threatening form of anaphylactic reaction. Nevertheless, understanding of its pathophysiology to support new therapies still needs to be improved. We performed a systematic review, assessing the role and the complex cellular interplay of mitochondria and oxidative stress during anaphylaxis, mast cell metabolism and degranulation. After presenting the main characteristics of anaphylaxis, the oxidant/antioxidant balance and mitochondrial functions, we focused this review on the involvement of mitochondria and oxidative stress in anaphylaxis. Then, we discussed the role of oxidative stress and mitochondria following mast cell stimulation by allergens, leading to degranulation, in order to further elucidate mechanistic pathways. Finally, we considered potential therapeutic interventions implementing these findings for the treatment of anaphylaxis. Experimental studies evaluated mainly cardiomyocyte metabolism during AS. Cardiac dysfunction was associated with left ventricle mitochondrial impairment and lipid peroxidation. Studies evaluating in vitro mast cell degranulation, following Immunoglobulin E (IgE) or non-IgE stimulation, revealed that mitochondrial respiratory complex integrity and membrane potential are crucial for mast cell degranulation. Antigen stimulation raises reactive oxygen species (ROS) production from nicotinamide adenine dinucleotide phosphate (NADPH) oxidases and mitochondria, leading to mast cell degranulation. Moreover, mast cell activation involved mitochondrial morphological changes and mitochondrial translocation to the cell surface near exocytosis sites. Interestingly, antioxidant administration reduced degranulation by lowering ROS levels. Altogether, these results highlight the crucial role of oxidative stress and mitochondria during anaphylaxis and mast cell degranulation. New therapeutics against anaphylaxis should probably target oxidative stress and mitochondria, in order to decrease anaphylaxis-induced systemic and major organ deleterious effects.

## 1. Introduction

Anaphylactic shock (AS) is the most severe form of immediate allergic hypersensitivity reaction, and is life-threatening [1]. Anaphylaxis is due to a prompt massive release of active mediators by inflammatory cells following allergen exposure. In most cases, a previous contact with the allergen yielded to sensitization. Although AS is rare, its exact incidence remains difficult to assess due to the various factors characterizing these events. The current definition of anaphylaxis is complex with a wide range of clinical presentations leading to under-reporting of allergy cases. Additionally, anaphylaxis has an acute and unexpected onset and may vary in severity, also leading to under-reporting of anaphylaxis cases. For all these reasons, epidemiological measures are likely to underestimate the incidence and prevalence of anaphylaxis. A systematic review suggest an incidence of 1.5–7.9 per 100,000 person-years, and prevalence is estimated at 0.3% (95% CI, 0.1–0.5) [2].

Pathophysiology of anaphylaxis and AS is usually summarized as immunologic or non-immunologic reactions. The mechanism most frequently encountered is an immunologic reaction mediated by immunoglobulin E (IgE) and involves mainly mast cells and basophils [3]. AS is commonly classified as a distributive shock (i.e., blood flow of organs, such as skeletal muscle, is decreased to preserve blood flow to vital organs, such as the brain and heart). However, the underlying cellular mechanisms are not fully understood. Dewachter et al. [4] described the cellular pathophysiology of AS in skeletal myocytes as an anaerobic metabolism related to a rapid decrease in partial skeletal muscle tissue oxygenation (PtiO_2_) without inhibition of the respiratory chain. The authors suggested a complete failure of energy production due to the decrease of oxygen availability in skeletal muscle. If this particular metabolism occurs in others cells and organs, it could result in rapid organ dysfunctions and explain the failure of well-conducted resuscitation. Using the same model of AS, our team observed an impairment in mitochondrial respiration in cardiomyocytes [5]. All together, these results supported the specific AS metabolism of different types of cells, partially due to a redistribution of regional blood flow, and could explain the occurrence of epinephrine-refractory AS cases. Mitochondria and oxidative stress are also largely involved in cellular metabolism of mast cells. Mitochondria increase adenosine triphosphate (ATP) production to enable mast cells to release anaphylactic mediators leading to clinical signs of AS and probably to impairment of mitochondrial function.

The description of mitochondrial and oxidative stress implications in AS is paramount to prevent cellular and organ energy depletion and altered function. Moreover, the understanding of the role of mitochondria in mast cell metabolism could lead to a new therapeutic strategy targeting mitochondria.

The aim of this systematic review was therefore to identify and analyze all relevant studies assessing the involvement of mitochondria and oxidative stress during anaphylaxis and mast cell degranulation. After presenting the main characteristics of anaphylaxis, the oxidant/antioxidant balance, and mitochondrial functions, we focused this review on the involvement of mitochondria and oxidative stress during anaphylaxis in animal model experiments. Then, we discussed the role of oxidative stress and mitochondria following mast cell stimulation by allergens leading to degranulation, in order to further elucidate mechanistic pathways. Finally, we considered potential therapeutic interventions implementing these findings for the treatment of anaphylaxis.

## 2. Anaphylaxis, Oxidative Stress and Mitochondria

### 2.1. Definition, Epidemiology, Pathophysiology, and Clinical Presentations of Anaphylaxis 

#### 2.1.1. Definition of Anaphylaxis

Hypersensitivity reactions are a significant concern for both the patient and their physician for medical care. Allergy is an abnormal immune response induced by an allergen. Hypersensitivity reactions are defined as dose-independent, unpredictable, harmful and unintended adverse effects following exposure to an allergen at a dose usually well-tolerated in humans [1]. Hypersensitivity reactions must be differentiated from toxic reactions. Immediate hypersensitivity reactions most often occur within an hour after contact with the allergen. Delayed hypersensitivity reactions usually begin several hours or days following allergen exposure.

According to the 2nd International Symposium on the Definition and Management of Anaphylaxis [6], anaphylaxis is a severe systemic reaction triggered by exposure to an antigen, characterized by respiratory and/or cardiovascular involvement, with cutaneous and/or mucosal involvement in most cases. American and European expert societies define anaphylaxis as a severe immediate systemic hypersensitivity reaction which is potentially life-threatening [7].

#### 2.1.2. Epidemiology of Anaphylaxis

The lifetime prevalence of anaphylaxis in the general population from all triggers has been estimated to be between 0.05 and 2% [8]. In the United States, the rate of severe anaphylaxis is increasing, with a 3.2-fold increase in anaphylaxis-related emergency department visits from 2008 to 2016 [9]. The incidence of anaphylaxis in Europe is estimated to be between 1.5 and 7.9 reactions per 100,000 person-years, with an increase in recent years. In the general population, anaphylactic reactions in children are primarily triggered by food allergens, whereas in adults, the main trigger is medication. Female patients seem to be at a higher risk of developing anaphylactic reactions [10,11]. Indeed, hormones such as estrogen or progesterone may act as immunomodulators, capable of increasing mast cell activation and thus triggering the anaphylactic reaction. Increasing age is both a factor that increases the incidence of anaphylactic reactions and the severity of such reactions, with a 2.35-fold increased risk after the age of 65 [12]. Finally, 71% of severe anaphylactic reactions resulting in patient death occur in individuals with a history of cardiovascular diseases [13].

#### 2.1.3. Anaphylaxis: A Complex Immune System Underlying Multiple Cellular Pathways

Immediate hypersensitivity reactions, which could lead to anaphylaxis and AS, depend on complex immunologic mechanisms [14]. Following exposure to allergens, their recognition by effector cells leads to a release of inflammatory mediators. Immediate hypersensitivity reactions are mediated by immunoglobulins of type E (IgE) or type G (IgG), and a direct activation of effector cells can lead to anaphylaxis.

Immunological mechanisms involve two immunoglobulin-mediated pathways. IgE-mediated anaphylaxis is the most frequently described physiopathological mechanism of anaphylactic reactions [15], which occurs in two phases. The first one is the sensitization phase, occurring after the first contact with the allergen, and is usually asymptomatic. Allergens are processed by antigen-presenting cells and presented via the major histocompatibility complex to T cells, which recruit B cells. This process triggers the production of antigen-specific IgE antibodies that bind to their specific high-affinity receptor, FcεRI, located on the surface of effector cells. The second phase is the effector phase, leading to clinical symptoms. Following a subsequent exposure to the allergen, the cross-linking of FcεRI-bound allergen-IgE complexes triggers the degranulation of basophils and mast cells and the massive release of vesicle contents [16].

The second Ig anaphylactic pathway is IgG-mediated. IgG are the most abundant immunoglobulins (85% of human serum immunoglobulins) and are secreted by plasma cells derived from B lymphocytes. They recognize an antigen to form an immune complex and bind to their FcγR receptor family. These receptors are located on the surface of many cells, such as neutrophil polymorphonuclear cells or mast cells. The IgG–receptor interaction activates the cell and releases inflammatory mediators. Hypersensitivity reactions mediated by IgG are described in the literature, but mainly in experimental mouse models. This IgG-mediated mechanism should explain some anaphylactic reactions associated with drugs, such as dextrans or aprotinin [17,18].

Basophils and mast cells can also release their mediators (histamine, tryptase, PAF) through a non-immunological pathway by direct contact with an allergen, without prior sensitization [19,20,21]. The allergens involved in this mechanism are primarily medications: opioids [22], antibiotics [23], neuromuscular blockers [24,25] or protamine [26]. The contact-anticoagulation system is also involved in anaphylaxis. The contact phase consists of the kinin-kallikrein system and the intrinsic pathway of coagulation. Activation of the contact phase is involved in thrombus formation and complement activation. Anaphylaxis mediators from mast cell degranulation (mainly heparin and polyphosphates) activate the contact phase by binding to and activating factor XII (factor XIIa). The intrinsic pathway is activated by factor XII, which activates both the contact system, and the coagulation system via factor XI, converting prothrombin to thrombin [27]. The end point of the coagulation cascade is clot formation. In addition to contact system activation, mast cell mediators may also be involved in the activation of the coagulation system.

Immediate hypersensitivity reactions mediated by the activation of Mas-related G-protein coupled receptor member X2 (MRGPRX2) expressed in mast cells has been recently described. The substances involved are primarily drugs such as neuromuscular blockers (atracurium, rocuronium) [28], antibiotics (fluoroquinolones [29], vancomycin [30]), or opioids. The binding of these substances to the MRGPRX2 receptor activates cytosolic G proteins, increases intracellular calcium concentration, and ultimately triggers the exocytosis of anaphylactic mediators [28].

Despite a thorough allergological work-up, no mechanism or causative allergen is identified in some clinical situations of anaphylaxis, which are therefore called “idiopathic anaphylaxis” [31]. Idiopathic anaphylaxis is a diagnosis of exclusion when no obvious etiological trigger explains clinical signs of anaphylaxis, affecting 30 to 60% of patients [32].

Thus, there are many experimental models related to the different physiopathological mechanisms leading to anaphylactic reactions. Numerous experimental models have been used to explain the mechanisms of anaphylactic reactions. In vitro studies have gradually given way to animal models, with an increasingly frequent use of genetically modified animals [33]. Indeed, the main advantage of experimental models is the high number of similarities in the immune system between rodents (mice) and humans [34].

Nevertheless, experimental models present limitations which affect clinical extrapolation to human physiology. It is unrealistic to consider only one type of physiopathological mechanism as the origin of an anaphylactic reaction. Furthermore, the immunization of animals is often based on large quantities of allergen compared to that of humans [35]. This large amount of antigen leads to both IgE- and IgG-mediated immunological anaphylactic reactions [3]. In humans, systemic anaphylactic reactions generally occur with a low quantity of antigens, which only favors an IgE-mediated response. They can also occur without an increase in serum IgE levels and without mast cell degranulation [34,36]. In humans, an IgG-mediated mechanism may evolve simultaneously with an IgE-mediated mechanism during anaphylactic reactions and can inhibit IgE-mediated clinical reactions [37]. This heterogeneity in the involvement of human mechanisms makes it difficult to use experimental models to explain the entire pathophysiology of anaphylaxis.

#### 2.1.4. Clinical Presentation of Anaphylaxis

The clinical presentation of anaphylactic reactions depends on various factors such as the allergen involved, the route of exposure, the presence of potential cofactors and the patient’s level of sensitization. The severity grade is usually based on the classification proposed by Ring and Messmer [38], which outlines four grades of increasing severity: grade I (mild clinical signs with exclusive cutaneous involvement), grade II (moderate multivisceral symptoms), grade III (life-threatening mono- or multivisceral symptoms), and grade IV (cardiorespiratory arrest). Clinical symptoms can affect all organs: urticaria and angioedema, digestive disorders with abdominal pain, nausea, vomiting, diarrhea, dyspnea, asthma or even bronchospasm, weakness, dizziness, confusion.

Symptoms of severe anaphylaxis may occur such as asphyxia, loss of consciousness and AS which can lead to cardiac failure and death [39]. More recently, the 2020 World Allergy Organization (WAO) anaphylaxis guidelines suggested a different systemic allergic reaction grading system [40]. In this five-grade classification, only grades 3, 4 and 5 would be consistent with the definition of anaphylaxis, and grades 4 and 5 correspond to AS. 

AS is usually described as a distributive shock with complex pathophysiology. Clinical signs may manifest as chest pain, tachycardia, arterial hypotension, vascular collapse, bronchoconstriction, or cardiac arrest. The massive release of vasoactive mediators, including histamine, platelet activating factor, nitric oxide, and arachidonic acid metabolites, leads to the effects on the cardiovascular system: vasoplegia, vascular hyperpermeability, and finally a profound hypovolemia. These mediators also affect bronchial smooth muscle, resulting in bronchoconstriction. A genuine cardiac dysfunction occurs during AS and results either from the direct action of anaphylactic mediators or from the hypovolemia and decrease in preload or from epinephrine side effects, including stress cardiomyopathy or Takotsubo syndrome.

In an experimental model of AS, some authors report regional blood flow impairments particularly in skeletal muscle or in brain with loss of cerebral autoregulation [4,41] and associated with splanchnic vasoconstriction [42] and pulmonary hypertension [43]. 

**Figure 1:**
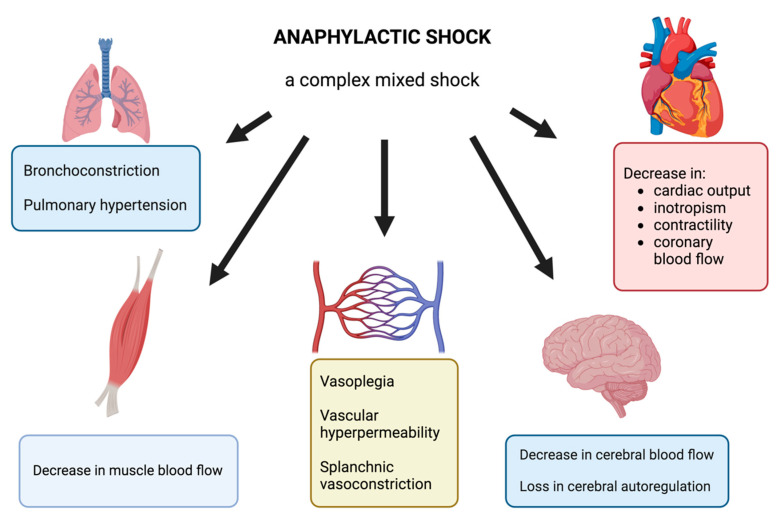
Illustration of the different organ dysfunctions during AS.

Based on the literature, Figure 1 summarizes the complex pathophysiology of AS and the different organ dysfunctions.

### 2.2. Oxidative Stress and Mitochondria

#### 2.2.1. Description of Mitochondria

**Figure 2:**
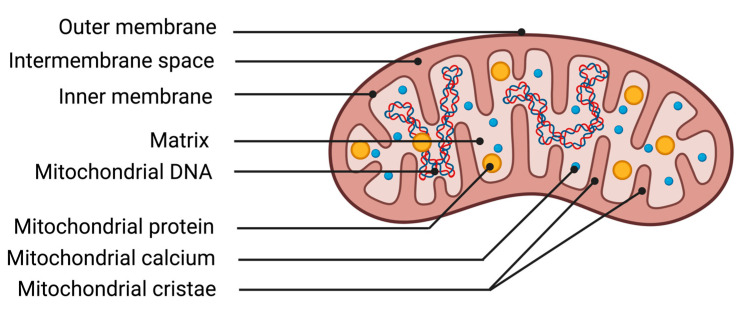
Illustration of mitochondrial structures. DNA deoxyribonucleic acid.

The mitochondrion is an organelle essential for cellular function due to its ability to transform energy. The protein complexes of the respiratory chain allow mitochondria to carry out their primary function, energy production through mitochondrial respiration. The chemical energy from substrates derived from nutrition, along with oxygen from respiration, is utilized by the mitochondrion to generate an electrochemical membrane gradient. This stored energy notably enables the synthesis of ATP, regulation of cellular calcium metabolism, protein transportation, and synthesis of macromolecules. Mitochondria are composed of different structures that delimit various compartments (Figure 2). The outer membrane is a lipid membrane primarily composed of transmembrane proteins called porins, which allow the passage of small molecules from the cytosol to the intermembrane space. The intermembrane space, localized between the outer and inner mitochondrial membranes, is the site of proton accumulation from the matrix during mitochondrial respiration, thereby creating an electrochemical gradient used for the synthesis of numerous molecules such as ATP. This space also houses several antioxidant systems essential for cellular survival during excess production of oxidative stress [44]. The inner membrane of the mitochondrion is a membrane composed of 75% proteins, with the remaining portion consisting of lipids. The enzymes of the mitochondrial respiratory chain are represented by five protein complexes anchored in the inner membrane. They enable the mitochondria to produce high-energy bonds (ATP molecules) through phosphorylating oxidation reactions. Finally, the matrix corresponds to the inner compartment of the mitochondria. The matrix is bordered by the inner membrane, which forms invaginations called mitochondrial cristae. Within the matrix are located all the enzymes necessary for the Krebs cycle and fatty acid beta-oxidation, as well as mitochondrial deoxyribonucleic acid (DNA).

Mitochondrial respiration or oxidative phosphorylation (OXPHOS) enables the mitochondria to produce energy in the form of ATP, which is essential for proper cell function. OXPHOS is linked to the enzymatic activity of five protein complexes located on the mitochondrial inner membrane. They enable the transfer of electrons from equivalent reducing molecules nicotinamide adenine dinucleotide (NADH) and dihydroflavine adenine dinucleotide (FADH_2_) (themselves from the Krebs cycle) to the final acceptor oxygen. Initially, complexes I and II transfer electrons from NADH and FADH_2_ respectively to complex III through the coenzyme Q or ubiquinone, a mobile component of the inner mitochondrial membrane. Then, complex III transfers the electrons to complex IV through cytochrome c. These electron transfers are coupled with proton transfers from the matrix to the intermembrane space. Complex V uses this electrochemical gradient to phosphorylate adenosine diphosphate (ADP) into ATP, by expelling protons into the matrix. 

Mitochondria are organelles which form a highly dynamic network. Within this dynamic state, we can distinguish three processes: mitochondrial fusion and fission; mitochondrial autophagy or mitophagy; and an active mitochondrial transfer in the cell. 

The processes of mitochondrial fusion and fission are very complex, are mediated by different proteins, and a fine balance between mitochondrial fusion and fission is crucial for cell survival and optimal functioning [45]. In mammals, mitochondrial fusion requires the involvement of two 85 kD-GTPase isoforms, namely mitofusin1 (Mfn1) and mitofusin2 (Mfn2), and another dynamin family 100 kD-GTPase, optic atrophy 1 (OPA1) [46,47]. Mfn1 and Mfn2 are anchored in the outer mitochondrial membrane, which mediates outer membrane fusion, while OPA1 is localized in the inner mitochondrial membrane and contributes to inner membrane fusion. Initial studies found that mitochondrial fission is regulated by the interaction of dynamin-related protein 1 (Drp1, also known as Dnm1l) and fission protein 1 (Fis1) [48,49]. Drp1 is mainly localized in the cytosol and Fis1 is anchored in the outer mitochondrial membrane.

To ensure a network of functional mitochondria, cells have quality control mechanisms in place that act at both the protein and organelle levels. Damaged mitochondrial proteins are degraded by proteases in the mitochondria and the ubiquitin-proteasome system [50]. If the degradation of damaged proteins is insufficient to rescue the mitochondrion, then the organelle is engulfed by an autophagosome and subsequently delivered to a lysosome for degradation. This selective removal of impaired mitochondria by autophagosomes is known as mitophagy. Both autophagy and mitophagy are critical for cellular homeostasis [51,52] and adapting to acute unfavorable conditions such as starvation or ischemia [53,54].

Apart from these intracellular mitochondrial dynamics, horizontal transfer of mitochondria inside and between mammalian cells was recently shown [55]. The diverse functionality of mitochondria has been linked to their morphological complexity. Additionally, mitochondria in many cell types form a complex reticulum by which they develop an intracellular mitochondrial transport. In the cell, the mitochondrial movement requires cytosolic motor proteins (kinesin, dynein, myosin) and a motor-adaptor complex which associates mitochondria to motor proteins [56]. The regulation of mitochondrial movement is mainly due to cytosolic calcium concentration. Elevation of cytosolic calcium stops both the anterograde and the retrograde movement of mitochondria.

#### 2.2.2. Definition of Oxidative Stress

In the cell, enzymatic reactions involve transfers of energy, electrons, or protons. Some molecules such as oxygen or nitrogen can capture or donate an electron, which becomes unpaired. This new molecule is called a “free radical” because it can interact with other more stable molecules. Reactive oxygen species (ROS) or reactive nitrogen species (RNS) contribute to physiological processes essential for proper cellular function or to pathophysiological processes leading to energy production impairment, apoptosis or mitophagy.

Oxidative stress can be defined as an imbalance between the production of ROS and the neutralization of these species by antioxidant systems.

#### 2.2.3. ROS Production

**Figure 3:**
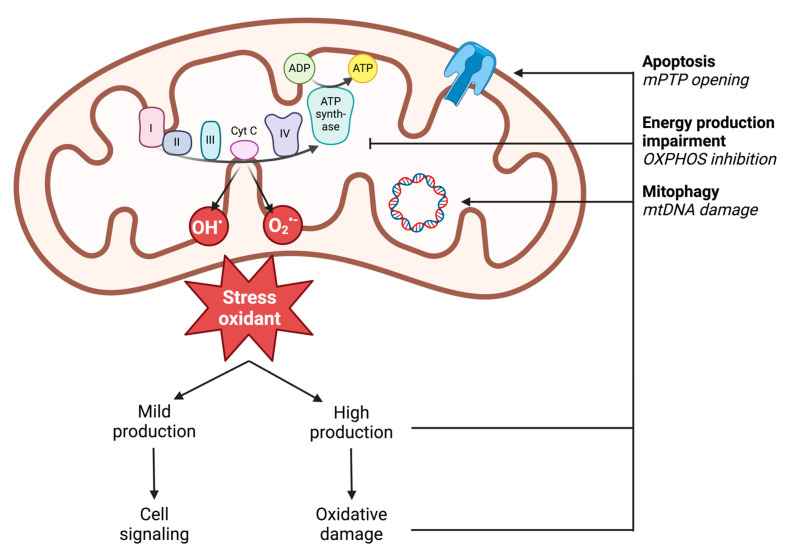
Illustration of mitochondrial ROS production and the deleterious effects of high production on mitochondrial functions. ADP: adenosine diphosphate; ATP: adenosine triphosphate; mPTP: mitochondrial permeability transition pore; mtDNA: mitochondrial DNA; O_2_^•−^: superoxide anion; OH^•^: hydroxyl radical; OXPHOS: oxidative phosphorylation; I: complex I; II: complex II; III: complex III; IV: complex IV; cyt c: cytochrome c.

In vivo, ROS production results from enzymatic and non-enzymatic reactions. Enzymatic production is linked to the activity of cellular enzymes. Nicotinamide adenine dinucleotide phosphate (NADPH) oxidases are localized on the outer cell membrane or within specific granules of polymorphonuclear cells, macrophages, and endothelial cells. Through the generation of ROS, notably superoxide anion (O2^•−^), NADPH oxidases enable immune cells to phagocytose and lyse pathogens or foreign molecules [57]. Additionally, enzymes in the arachidonic acid synthesis pathway, peroxisomes, or lysosomes can produce superoxide radicals (O2^•−^) [58]. However, non-enzymatic production is the main source of cellular ROS and comes from the mitochondrial respiratory chain through the five complexes of the mitochondrial respiratory chain (Figure 3) [59]. Accordingly, in settings with similar reduction in perfusion like during ischemia-reperfusion, impaired muscle mitochondrial respiration is related to oxidative stress with increased ROS production potentially blunted by the antioxidant defense of the cells damaged [60,61]. Under physiological conditions, 0.2 to 2% of the electrons generated from the substrate oxidation activity leak out of the respiratory chain and interact directly with oxygen to produce ROS [62]. However, different mitochondrial sites, such as enzymatic complexes (2-oxoglutarate dehydrogenase or pyruvate dehydrogenase), are also capable of considerable ROS production depending on the substrate present [63]. The remaining sites involve mitochondrial matrix dehydrogenases. The capacity of these three complexes to produce ROS decreases in the following order: Complex I > Complex III > Complex II [59,64].

Among the reactive oxygen species, the following are distinguished:

- Primary free radicals resulting directly from the reduction of oxygen. Example: superoxide anion O2^•−^, hydroxyl radical OH^•^.
- Secondary free radicals formed by the reaction of primary free radicals with cellular biochemical compounds. Example: reaction of superoxide anion O2^•−^ with nitric oxide to form peroxynitrite ONOO^−^.
- Active oxygen species: these molecules do not possess unpaired electrons but have a strong oxidizing power as they can generate free radicals. Example: hydrogen peroxide H_2_O_2_.

#### 2.2.4. Deleterious Effects of ROS: Oxidative Damage

The production of oxidative stress is mainly linked to the functioning of the mitochondrial respiratory chain, resulting in the production of radical species derived from oxygen such as O2^•−^ and OH^•^ or derived from nitrogen as peroxynitrite (ONOO^−^) or non-radical molecules (H_2_O_2_).

These molecules react rapidly with molecules close to their production site and cause oxidative damage to the cell. This oxidative damage is defined by three categories of damage [65,66,67]: lipid peroxidation, protein oxidation, and DNA mutations. This damage can lead to loss of protein function, cell integrity, and even cell death by apoptosis.

Oxidative stress molecules can induce oxidative damage to lipids, known as lipid peroxidation, which can impair cellular function. Oxidative stress can lead to lipid peroxidation either through radical molecules such as O2^•−^ or HO^•^ [66], or through non-radical molecules like H_2_O_2_ [68]. Consequently, lipid peroxidation can directly affect cellular or mitochondrial membrane lipids, leading to loss of cellular or mitochondrial function, or indirectly by increasing membrane permeability to H_2_O_2_, thus enhancing its cellular toxicity [69]. Lipid peroxidation may result in a loss of function of mitochondrial cardiolipin [70], a major and specific component of the inner membrane. The phospholipid cardiolipin is a key regulator of various mitochondrial functions [70]: protein import into mitochondria, transfer of cytosolic NADH to the mitochondrial respiratory chain, and stability and optimal enzymatic activity of respiratory complexes. Proteins involved in the respiratory chain are also affected by lipid peroxidation [71] through decreased activity of complexes I to V or enhancement of uncoupling, resulting in decreased ATP production.

Based on the results of this systematic review, protein oxidation and DNA mutations are not involved in anaphylactic pathophysiology. Thus, we did not describe these types of oxidative damage.

## 3. Methods of the Systematic Review of the Literature

The stepwise systematic review process was performed according to the Preferred Reporting Items for Systematic Reviews and Meta-analyses (PRISMA) guidelines [72]. Two authors (AP and WO) screened all references and independently evaluated every study. A senior third author (BG) settled any discrepancies, and articles were evaluated to reach a consensus statement and open perspectives.

### 3.1. Selection Criteria

Inclusion and exclusion criteria were followed throughout the literature screening process to identify relevant studies. All references investigating the relationship between anaphylaxis or mast cell degranulation, and mitochondria or oxidative stress were included in the analysis. There was no limitation regarding the date of publication. Reviews, editorials, congress abstracts, and non-English articles were excluded. Studies that described the experimental model used, the stimulation used for inducing degranulation, the assessment of in vivo anaphylaxis or in vitro mast cell degranulation, and assessment of oxidative stress and mitochondrial function were considered to have sufficient data. References for which the full text could not retrieved were not included.

### 3.2. Research Strategy and Data Collection Process

The MEDLINE database (via PubMed) was analyzed to retrieve eligible studies. We performed preliminary searches to identify key words highlighted in articles dealing with the topic. The most relevant keywords to the review’s objective were discussed among the authors to reach a consensus. We then ensured that relevant articles could be found in the literature with the final terms chosen. The search strategy was based on the following terms: ‘mitochondria’, ‘mitochondrial respiration’, ‘oxidative stress’, ‘mast cell degranulation’, ‘anaphylaxis’, ‘anaphylactic shock’ and ‘allergic shock’. Each individual term was combined with at least one other term following a rational approach (see Supplementary S1 in Supplementary Materials). The search has been updated up to 22 March 2024.

A flow chart was generated step by step, reporting documented and excluded articles throughout the systematic review process.

After removal of duplicate records, titles and abstracts of each reference were screened for relevance. Eligibility criteria were applied, and the full texts of selected references were retrieved. References sections of selected papers were screened to identify any additional relevant studies. Articles that have cited selected studies were analyzed to find other potentially relevant papers. Final evaluation based on full-text manuscripts was carried out for inclusion in the systematic review.

All outcomes were defined on a standardized set of information extracted from each study: assessment of mitochondrial respiration reactive oxygen species, mitochondrial DNA, mitochondrial particles, mitochondrial and cytosolic calcium flows, lipid peroxidation and mitochondrial dynamic. Other variables for which data were sought included first author, publication year, animal model, cells stimulated, anaphylaxis model, model to induce mast cell degranulation, assessment of anaphylaxis and mast cell degranulation. All data collected for each study were coded in a standardized Excel sheet independently by two authors to reduce biased assessment.

For the synthesis, studies on animal models (in vivo and ex vivo) and studies on cellular models (in vitro) were presented separately. Within the review of studies on cellular models, results were described according to the mechanistic pathways investigated.

## 4. Results

### 4.1. Study Selection

**Figure 4:**
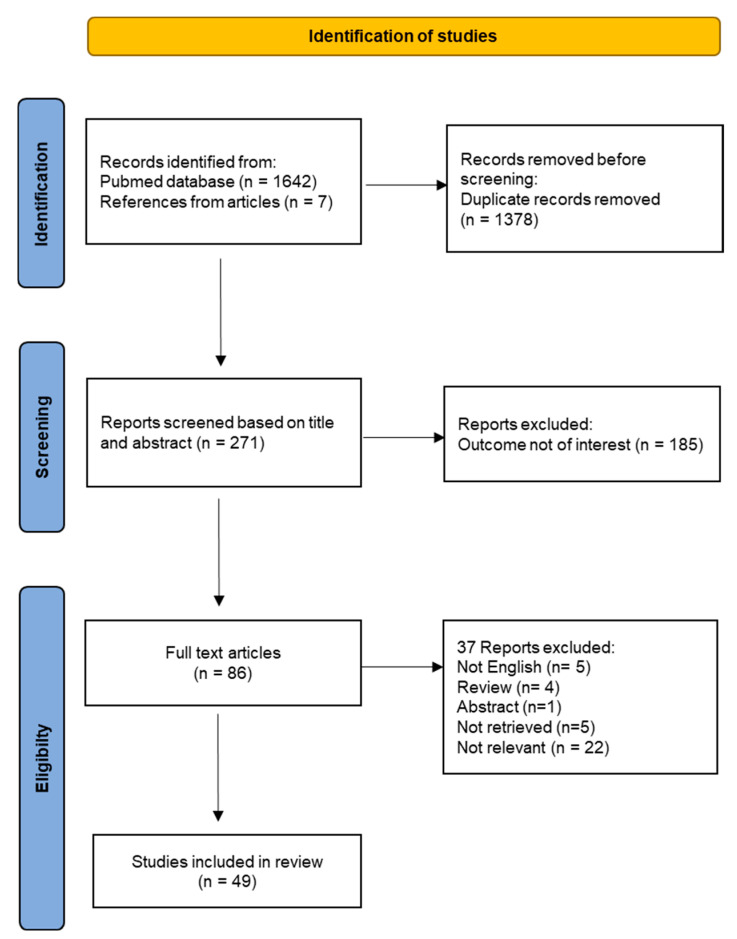
Flow-chart of the systematic review.

The flow chart for the study selection is provided in Figure 4. A total of 271 unique titles and abstracts were evaluated, from which 185 references were excluded because they were irrelevant to the systematic review topic. The remaining 86 full-text articles were analyzed for eligibility. Out of those, 15 articles were excluded because they do not meet selection criteria and 22 articles were removed because the outcome was not relevant. Finally, the full review process yielded a total number of 49 studies included in the systematic review.

### 4.2. Study Characteristics

Twenty-one studies were carried out on animal models, of which 18 were conducted in vivo and three ex vivo. Most studies have used murine animal models, the remainder have used pigs. These studies used active or passive anaphylactic models. Assessment of anaphylaxis was based on temperature, blood pressure, heart rate, blood flow, cardiac function, and airway bronchoconstriction or passive cutaneous anaphylaxis (PCA) reaction. The PCA model is a widely used model to assess IgE-mediated anaphylaxis [73], based on the assessment of the degree of extravasation of Evans blue dye which reflects a vascular leakage following mast cell activation and anaphylaxis. Laboratory analyses were performed on blood cells, cardiomyocytes, hepatocytes, and subcutaneous air-pouch lavage fluid cells. Among the 21 articles, only nine proposed cellular pathways involving oxidative stress and mitochondria to explain anaphylactic signs. The remaining 12 studies explored the effects of various intervention effects on mast cell degranulation in vitro, and used animal models only to assess the occurrence of anaphylaxis in vivo.

A total of 40 studies analyzed in vitro mast cell degranulation. Studies were performed on various mast cell lines: antigen-stimulated rat basophilic leukemia cells (RBL2H3), bone marrow mast cells (BMMCs), fetal skin-derived mast cells (FSMCs), human leukemic LAD2 mast cells, human umbilical cord blood–derived mast cells (hCBMCs), human leucocytes, human peripheral blood–derived cultured mast cells (HCMCs), human skin mast cells, mouse peritoneal mast cells, and primary human keratinocytes. Sixteen studies used an IgE-mediated mast cell stimulation method, four studies used a non-IgE method, and 10 studies assessed both methods of stimulation. Mast cell degranulation was usually assessed by β-hexosaminidase, serotonin and histamine release.

### 4.3. Mitochondrial Function and Mast Cell Degranulation

There is a link between mast cell degranulation and the mitochondrial respiratory complex chain activity resulting in oxidative phosphorylation. Usually, activation of mast cells is associated with an enhanced mitochondrial respiration. Thus, IgE-mediated activation induced OCR to increase by 60% [74]. Accordingly, mitochondrial respiratory complex chain inhibition reduced IgE- and non-IgE-mediated mast cell degranulation. Oligomycin was shown to decrease IgE- and non-IgE-mediated mast cell degranulation [75,76], but it was not confirmed by another study [77]. Inhibition of complex I and III reduced IgE-mediated mast cell degranulation [75,78]. Inhibition of complex I and II decreased non-IgE mediated degranulation [75,79]. However, inhibition of complex III did not decrease IgE mast cell degranulation in some studies [74,79].

The mitochondrial membrane potential generated by the electron transport chain (ETC) modulates mast cell degranulation. OXPHOS uncoupling has been shown to decrease mast cell degranulation. Collapse of potential mitochondrial membrane induced by the uncoupler carbonyl cyanide-4-(trifluoromethoxy)phenylhydrazone (FCCP) reduced mast cell degranulation after IgE and non-IgE mast cell stimulation [75,80]. It should be one of the operating modes of Triclosan to decrease IgE-mediated mast cell degranulation [81]. However, FCCP, in the absence of extracellular Ca^2+^, can promote degranulation through rapid mitochondrial depolarization and release of stored Ca^2+^ [75].

Furthermore, most of the energy for mast cell degranulation comes from mitochondrial ATP. Indeed, 5 min after non-IgE stimulation of mast cells, mitochondrial ATP level increases by nearly 40% [82]. Triclosan inhibits IgE and non-IgE mast cell degranulation by decreasing ATP production [83,84].

Upstream of ETC, the pyruvate dehydrogenase (PDH), which is the key regulator for NADH production and TCA cycle activity, also proved its important role for mast cell degranulation. Indeed, PHD inhibition led to significant reduction in ATP production correlated with a reduction of mast cell degranulation [85]. These results were also supported in a mouse model with a decrease of anaphylaxis and histamine release after PDH inhibition [85].

## 5. Discussion

### 5.1. Experimental Studies in Animal Models

Experimental studies in animal models of our systematic review concerning oxidative stress and mitochondria during anaphylaxis are summarized in Table 1. In two recent studies, our team reported reproducible clinical signs in an experimental model of AS [5,86]. Animals presented a severe and rapid decrease in mean arterial pressure, cardiac output and contractility within 5 min after AS onset. All animals died without treatment after 20 min. While a cardiac dysfunction occurred during AS, we observed a cardiac mitochondrial function impairment with a 40% decrease in maximal OXPHOS state and a 30% increase in cardiac lipid peroxidation associated with a trend towards increase in oxidative stress (+25% peroxynitrite). At the same time, we observed an increase in cardiac superoxide dismutase (SOD) activity (+40%, *p* < 0.05). These results may indicate that cardiac oxidative damage and mitochondrial respiration impairment participate in cardiac dysfunction during AS despite the involvement of the antioxidant system. In another study using the same AS model, Bellou et al. observed similar hemodynamic dysfunctions with a severe and rapid decrease in mean arterial pressure (MAP) in shocked rats without treatment [87]. The authors did not explain their clinical findings by underlying cellular mechanisms. They reported also an increase in SOD plasma level in the AS group, whereas catalase activity and thiobarbituric acid reactive substances (TBARs) plasma level were not modified. Increase in oxidative stress during anaphylaxis was confirm by Kloek et al. [88]. The authors observed an increase in lipid peroxidation (increase in TBARs production) in the respiratory tract in an IgG-mediated model of AS using ovalbumin-sensitized guinea pigs. In an AS model of peanut allergy using mice sensitized with peanut extract, Trinchese et al. [89] analyzed hepatic mitochondrial dysfunction. Despite a higher SOD activity (+26%, *p* < 0.05), they observed an increase in oxidative stress characterized by an increase of 52% in H_2_O_2_ (*p* < 0.05) resulting from inactivation of aconitase, a Krebs cycle enzyme highly sensitive to oxidative stress.. Concerning mitochondrial respiration, the authors reported a decrease in OXPHOS through complexes I and II. Milicic et al. reported a decrease in cardiac oxidative stress during AS [90]. In an ex vivo model of AS, the authors observed within 2 min of AS a decrease in lipid peroxidation assessed by TBARs production, and no change in oxidative stress by superoxide anion radical (O2^•−^) assessment in samples of coronary effluent collected at different times after AS onset. After testing the iNOS/NO pathway with genetically modified mice, the authors concluded that this pathway may not be the only influential mediator of cardiac anaphylaxis. In a similar model of AS using ovalbumin-sensitized guinea pigs, Rosic et al. [91] noticed an increase of cardiac ROS (O2^•−^ and H_2_O_2_) release during anaphylaxis. They did not observe any change in NO concentration before, during or after ovalbumin administration in coronary effluent, suggesting that the iNOS/NO pathway may not be involved in cardiac anaphylaxis.

Concerning mitochondrial structural alteration, previous in vivo studies showed morphological modifications of mitochondria during anaphylaxis and especially during AS. In 1972, Suzuki et al. [92] observed immediate ultrastructural changes in cardiac mitochondria in an IgE-mediated model of AS. The authors described shrunken or disrupted cardiac mitochondria with close cristae and dense matrix. In 1985, Dimlich et al. [93] observed a significant decrease in mitochondrial granules of hepatocytes of rats subjected to compound 48/80-induced AS, probably due to a loss of intramitochondrial calcium as occurs in epinephrine-induced glycogenolysis in the rat. In the same way, we did not report any change in cardiac mitochondrial ultrastructure within 15 min after AS onset, whereas hemodynamic and cardiac mitochondrial function were severely impaired [5].

**Figure 5:**
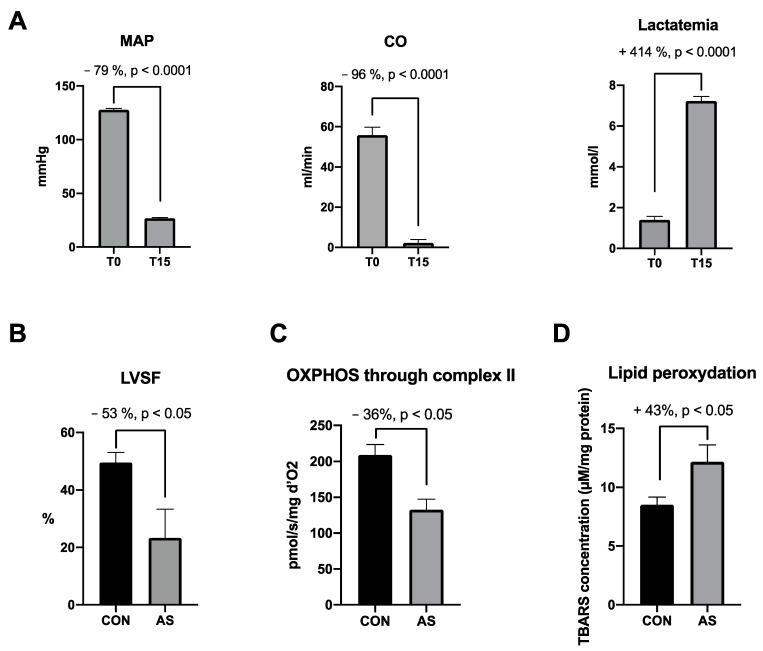
Major effects of AS on hemodynamic, cardiac and mitochondrial functions focused on cardiac dysfunctions, selected from the literature. (**A**) Hemodynamic impairments and increase in lactatemia in AS group between T0 and T15 min [5,94]. At T15 min we observe in AS group a (**B**) cardiac contractility decrease [86], a (**C**) cardiac mitochondrial impairment through complex II [5,86] and a (**D**) cardiac lipid peroxidation increase [5] as compared with CON group. MAP: mean arterial pressure, CO: cardiac output, LVSF: left ventricle shortening fraction, OXPHOS: oxidative phosphorylation, CON: control group, AS: shocked group, TBARs: thiobarbituric acid reactive substances.

Altogether, AS was characterized by a severe drop in systemic pressure and cardiac output associated with a significant increase in lactatemia (Figure 5A). The cardiac dysfunction is associated with the cardiac mitochondrial dysfunction occurring within the first minutes of AS. Fifteen minutes following AS induction, a decrease in cardiac contractility was associated with a significant cardiac mitochondrial respiration impairment and cardiac lipid peroxidation (Figure 5B–D).

### 5.2. Cellular Studies

Mast cells are major actors inducing anaphylaxis through a massive release of inflammatory mediators after contact with a specific antigen. It is well established that mast cells produce ROS after stimulation with antigen and non-FceRI stimuli such as substance P or compound 48/80 [95,96]. Experimental data are summarized in Table 2 and Table 3.

#### 5.2.1. Relation between Mast Cell Degranulation and Mitochondrial Function

**Figure 6:**
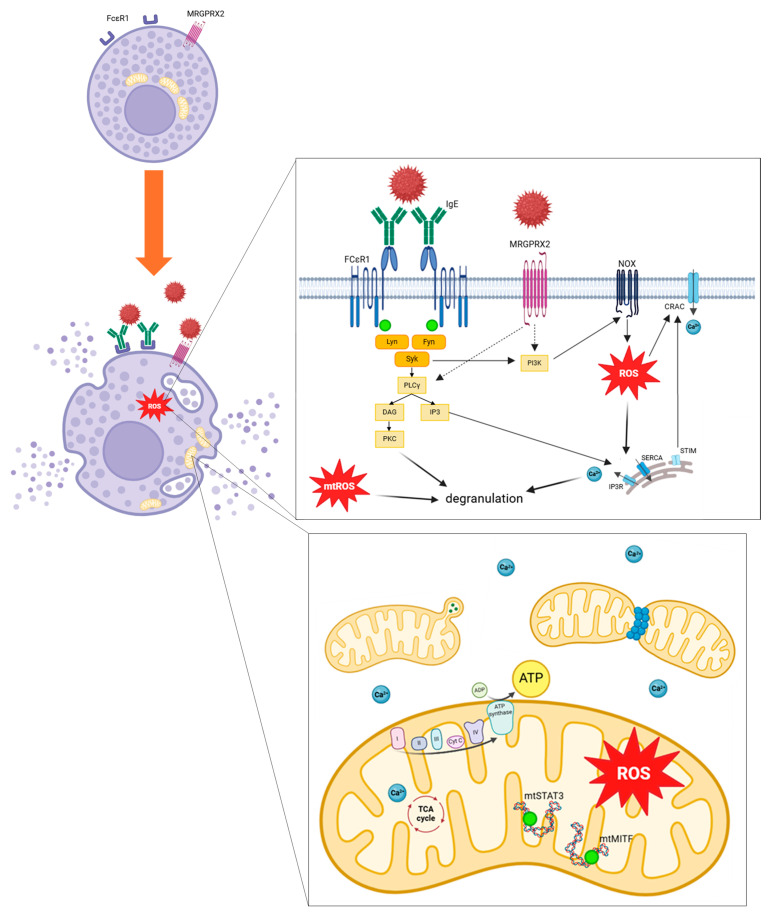
Mitochondrial involvement and oxidative stress pathways implicated during mast cell stimulation. ADP, adenosine diphosphate; ATP, adenosine triphosphate; Ca^2+^, calcium ion; CRAC, calcium release-activated channels; cyt c, cytochrome c; DAG, Diacylglycerol; FcεRI, high-affinity IgE receptor; Fyn, Src family kinase; I mitochondrial complex I; II mitochondrial complex II; III mitochondrial complex III; IV mitochondrial complex IV; IgE, immunoglobulin E; IP3, Inositol 1,4,5,-triphosphate; IP3R, inositol trisphosphate receptor; Lyn, Src family kinase; mtSTAT3, mitochondrial signal transducer and activator of transcription 3; mtMITF, mitochondrial microphthalmia associated-transcription factor; MRGPRX2, Mas-related G-protein coupled receptor member X2; mtROS, mitochondrial ROS; NOX, NADPH oxidase; PLCγ, phospholipase Cγ; PI3K, Phosphatidylinositol 3 kinase; PKC, Protein kinase C; ROS, Reactive Oxygen Species; SERCA, sarco/endoplasmic Ca^2+^-ATPase; Syk, Spleen tyrosine kinase; STIM, stromal interaction molecule; Syk, Spleen tyrosine kinase; TCA cycle, tricarboxylic acid cycle.

As previously detailed, mitochondrial respiratory complex chain integrity and mitochondrial membrane potential are essential for optimal mast cell degranulation. IgE and non-IgE mast cell stimulation tends to enhance OXPHOS through complexes I and III, leading to increased mitochondrial ATP production and allowing degranulation. More precisely, after allergen stimulation through IgE receptor FcεRI, activation of Lyn, Fyn and Syk kinases promotes activation of PLCγ and PI3K. PLCγ generates DAG and IP3, leading to calcium-dependent degranulation. IP3 binds to its receptors IP3R on endoplasmic reticulum and induces Ca^2+^ release. PI3K promotes ROS production through NOX activity. MRGPRX2 activation should promotes PI3K and PLCγ activation. Increase in intracellular Ca^2+^ induces granules exocytosis. ROS regulates mast cell degranulation through Ca^2+^ flux from endoplasmic reticulum and extracellular influx by CRAC. mtROS regulates PKC phosphorylation, leading to degranulation. Mast cell activation induces mitochondrial fragmentation, morphological changes and translocation of mitochondria from perinuclear region to the cell surface. Phosphorylation of the two transcriptional factors STAT3 and MITF induces mitochondrial respiration, production of ATP and mitochondrial ROS, promoting mast cell degranulation (Figure 6).

#### 5.2.2. Relation between Mast Cell Degranulation and Oxidative Stress

ROS have a key role in mast cell regulation, including activation and degranulation.

In an ex vivo model of cardiac anaphylaxis in pigs, a significant increase of O2^•−^ and H_2_O_2_ blood concentration was observed during allergen challenge compared to baseline values [91]. There was also a higher mitochondrial H_2_O_2_ production in liver from mice sensitized with allergen compared to control group after allergen challenge, despite a higher SOD activity [89]. However, these data were not confirmed in an ex vivo model of cardiac anaphylaxis in isolated mice hearts in which H_2_O_2_ blood concentration decreased after allergen challenge [90].

Non-IgE mast cell stimulation by sodium sulfite induced ROS generation and mast cell degranulation in a dose-dependent manner [79,99]. Inhibition of mtROS generation or decrease of intracellular ROS levels suppressed histamine release independently from IgE stimuli [79]. In the same way, IgE stimulation of mast cells led to increased intracellular ROS levels and degranulation [97,104]. After allergen stimulation, intracellular ROS production in mast cells increased in a time- and allergen dose-dependent manner before reaching a plateau [100]. Interestingly, IgE-induced ROS production seemed to be more rapid and of less amplitude than non-IgE-induced ROS production (by thapsigargin). This latter appeared entirely due to intracellular Ca^2+^ influx while allergen-induced ROS generation should be at least partly due to NADPH oxidase activity [101].

FCER1 stimulation induced intracellular ROS production, mediated by NADPH oxidase activity through phosphatidylinositol 3 kinase (PI3K) [98,102]. Rotenone did not inhibit oxidative burst, suggesting that IgE-induced ROS production is mostly mediated by NADPH oxidase activity [95]. Furthermore, tyrosine phosphorylation of PLCγ is ROS-dependent, suggesting a potentiating role of ROS production in allergic signaling pathways [98]. Hydrogen treatment of mast cells downregulates NADPH oxidase activity and consequently reduces ROS production and β-hexosaminidase release in allergen-stimulated mast cells [102]. Furthermore, inhibition of NADPH oxidase also suppressed antigen-induced phosphorylation of Lyn, a member of the Src family of protein tyrosine kinases and downstream targets [102]. This suggests that NADPH oxidase activity and its intracellular ROS production are involved in FCER1-mediated signal transduction and allergen-induced mast cell degranulation as a feed-forward loop that potentiates the allergic response. These results were confirmed in a mouse model of passive cutaneous anaphylaxis. Pre-treatment with hydrogen-rich water decreased leakage of Evan’s blue dye and reduced serum histamine release compared to control mice [102].

MtROS-induced histamine release was decreased by protein kinase C (PKC) inhibitor, while depletion of intracellular Ca^2+^ had no effect, suggesting that mtROS modulates PKC activity for release of biogenic amines from mast cells [79]. Furthermore, in the presence of mtROS in mast cells, there was no significant enhancement of Annexin V binding, which is required for granules exocytosis, compared to antigen-stimulated mast cells. This suggests that mtROS induces signals for granules migration, but not sufficiently for the release of granules’ total content [79].

Furthermore, overexpression of uncoupling protein 2 (UCP2) reduced mast cell degranulation, and inhibition of UCP2 enhanced histamine release and vascular permeability in mice after allergic and non-allergic stimulation [103].

Moreover, biogenic amines (histamine and serotonin) are positively-charged low molecular weight molecules which can be release through an increase of an alkalization of secretory vesicles. Increase in intraluminal pH of secretory vesicles was observed in mast cells with mitochondrial dysfunction, and inhibition of vacuolar H+-ATPase led to increased histamine release. This suggests that mtROS could be implicated in an inhibition of vacuolar H+-ATPase in secretory granules, leading to release of biogenic amines (histamine and serotonin) from mast cells [79].

Environmental pro-oxidant conditions could enhance mast cell degranulation. Indeed, exposure to environmental fine particulate matter (PM2.5), one the major scourges involved in indoor and outdoor air pollution, could facilitate IgE mast cell activation, as it increased ROS production, up-regulated manganese (Mn)-SOD activity, and upregulated β-hexosaminidase release and cytokine production from mast cells following allergen stimulation. It was supported by enhancement of Evan’s blue extravasation and cytokine production in a mouse model of passive cutaneous anaphylaxis [108]. Acrolein, one of the main sources of urban air pollution found in tobacco smoke and combustion products, also demonstrated its potential involvement in allergic disease through promoting mast cell degranulation. Indeed, acrolein induces intracellular ROS generation in a dose-dependent manner and degranulation in mast cells [105].

In summary, these studies indicate that antigen stimulation of mast cells leads to increased ROS production from NADPH oxidases and from the mitochondrial respiratory chain (Figure 6). In vitro antioxidant treatment of mast cells seemed to reduce degranulation through decreased ROS production, but further studies are needed to confirm it. Exposure to pro-oxidant environmental conditions such as air pollution seems to enhance mast cell degranulation by increasing ROS production. Selected studies investigating mast cell degranulation in relation with oxidative stress and lipid peroxidation are presented in Table 2.

#### 5.2.3. Mitochondrial Transcription Pathways

The pool of the two transcription factors microphthalmia-associated-transcription factor (MITF) and signal transducer and activator of transcription 3 (STAT3) localized in mast cell mitochondria are involved in mitochondrial respiration, ROS production and mast cell degranulation.

MAPKs signaling pathways activation following mast cell stimulation leads to mitochondrial STAT3 phosphorylation by ERK1/2 on serine 727, which has a key role in initiating ATP production by OXPHOS during mast cell activation [82]. In a mouse model of passive systemic IgE-mediated anaphylaxis, histamine release was reduced by 43% by inhibiting STAT3 [82].

Treatment of mast cells with the mitochondria-targeted inhibitors Mitocur-1 and Mitocur-3 reduced mitochondrial ATP levels in a dose-dependent manner, mitochondrial oxygen consumption rate, and subsequent degranulation after IgE mast cell stimulation [118]. Pre-treatment of activated mast cells with Mitocur 1/3 reduced STAT phosphorylation on Ser 727 leading to reduced allergen-dependent degranulation, but Mitocur1/3 did not affect unstimulated mast cells and spontaneous degranulation [110]. Moreover, mice treated with Mitocur 1 before IgE-induced anaphylaxis had reduced plasmatic histamine levels [118].

The polyphenol Resveratrol was shown to inhibit mast cell degranulation through inhibiting ERK1/2 activation, and subsequent mitochondrial STAT3 activation [121]. Furthermore, PIAS3, the main endogenous inhibitor of STAT3, localized in mitochondria, was also increased after mast cell activation and acted as a regulator of mitochondrial allergic response [82].

MITF is a key regulator of mast cells by interacting in the E1 subunit of PDH. Indeed, PDH have a major role in OXPHOS activity which is dependent on NADH and FADH2 produced during the TCA cycle. Mast cell activation led to dephosphorylation of PDH, phosphorylation on MITF, detachment of MITF from the E1 subunit of PDH, enhancement of PDH activity, and increased OXPHOS activity, ATP production, degranulation, and cytokine production in antigen-stimulated models [85,122].

Mast cell activation through MRGPRX2 leading to ERK1/2 activation signaling pathways increased MITF phosphorylation and activation [124]. MITF silencing reduced β-hexosaminidase release and CD63 expression from mast cells [124]. Furthermore, drug activation of MRGPRX2 by vancomycin, atracurium, morphine and meglumine amidotrizoate increased MITF activity in mast cells [124].

In summary, phosphorylation of mitochondrial transcription factors STAT3 and MITF after antigen stimulation of mast cells led to increased OXPHOS activity, ATP production and degranulation (Figure 6).

#### 5.2.4. Role of Mitochondria on Ca^2+^ Flux during Mast Cell Degranulation

Ca^2+^ homeostasis is crucial for cell activity and viability. Mitochondria appear to play a buffering role by controlling Ca^2+^ flux. The mitochondrial Ca^2+^ level is essential for activation of Ca^2+^-sensitive dehydrogenase of the TCA cycle, mitochondrial respiration, and ATP production. Nevertheless, mitochondrial Ca^2+^ overload can lead to excessive ROS production and impair bioenergetic metabolism and cell viability.

Mast cell activation through IgE or non-IgE pathways induces cytosolic Ca^2+^ increase [75].

Ca^2+^ release from the endoplasmic reticulum (ER) induced by inositol triphosphate (IP3) and stromal interaction molecule 1 (STIM1) mediated store-operated entry (SOCE) are involved in cytosolic Ca^2+^ level increase following IgE mast cell stimulation [78].

Mitochondrial calcium uniporter (MCU) is also required for mitochondrial Ca^2+^ buffering capacity. Mitochondrial Ca^2+^ uptake and degranulation was reduced by MCU knockdown in IgE and non-IgE stimulated mast cells [115,116]. Allergen stimulation of mast cells led to increased mitochondrial Ca^2+^ via MCU more rapidly and more persistently correlated with antigen concentration [112].

Moreover, mitochondrial Ca^2+^ efflux seemed to be mediated by mitochondrial transition pore (mPTP) rather than mtNCX [112]. IgE-induced Ca^2+^ release in cytosol was enhanced by mitochondrial depolarization and dependent on mPTP (86). Mitochondrial ROS seemed to enhance mPTP sensitivity to mitochondrial Ca^2+^ leading to mPTP opening, cytosolic Ca^2+^ release, and subsequent mast cell degranulation [75]. Interestingly, non-IgE mast cell activation by thapsigargin induced less cytosolic Ca^2+^ increase in the absence of extracellular Ca^2+^ (and the contrary in the presence of extracellular Ca^2+^) suggesting IgE activation induced more Ca^2+^ store release [75].

Interestingly, it seems that antigen stimulation should increase mitochondrial Ca^2+^ rapidly and then induce slow Ca^2+^ level oscillations [84]. Triclosan seemed to disrupt mitochondrial Ca^2+^ buffering capacity by abolishing Ca^2+^ oscillations and inhibiting subsequent mast cell degranulation [84].

In addition, *Forsythiae fractus* (FRE), a dried fruit from traditional Chinese medicine with antioxidant properties reduced IgE and non-IgE mast cell degranulation and in vivo mouse IgE and non-IgE-mediated anaphylaxis through decreasing cytosolic Ca^2+^ [120]. The antimicrobial Cetylpyridinium chloride (CPC) decreased IgE-mediated mast cell degranulation through decreasing Ca^2+^ efflux from RE and decreasing mitochondrial Ca^2+^ uptake into mitochondria [123].

In summary, calcium signaling, which regulates mast cell degranulation, is closely linked to mitochondrial functions. Mitochondria appear to play a buffering role in calcium cytosolic concentration (Figure 6). Alteration of mitochondrial buffering Ca^2+^ capacity induces a decrease in mast cell degranulation. In vitro studies investigating mast cell degranulation in relation with mitochondrial respiration, mitochondrial transcription factors and mtDNA, calcium flux, and mitochondrial dynamic and morphology are presented in Table 3.

#### 5.2.5. Cellular Consequences of Anaphylaxis-Associated Oxidative Stress

Although lipid peroxidation, measured by concentration of TBARs, should be enhanced during anaphylaxis in vivo [5,47], few studies analyzed lipid peroxidation during mast cell stimulation in vitro. Mitocur 1 and 3, inhibitors of STAT, increased cardiolipid peroxidation [110] as well as epigallocatechin-3-O-gallate (EGCG) [80]. Further explorations seem necessary to analyze molecular pathways involved in lipid peroxidation after allergen stimulation.

#### 5.2.6. Mitochondrial Morphology and Dynamics

Mitochondrial structure, morphology and dynamic changes occur during mast cell activation and degranulation.

Similar to the porcine model of IgE-mediated anaphylaxis, in vitro IgE activation of mast cells led to mitochondrial morphological changes and densely connected inner membranes with highly folded cristae [117].

Activation of mast cells through IgE or non-IgE stimuli led to mitochondrial fission and translocation from the perinuclear region to the cell surface [76,113,114,117]. Mast cell treatment with triclosan inhibited degranulation through inhibiting mitochondrial translocation in antigen-stimulated mast cells and inducing modification in mitochondrial morphology [81,84].

The mitochondrial translocation depends on Drp1 activity. Indeed, inhibition of Drp1 inhibited mitochondrial translocation and subsequent mast cell degranulation [113]. Thus, although still under debate, targeting the mitochondrial dynamic might be useful in situations characterized by organ hypoperfusion, like during severe anaphylaxis with reduced organ blood flow [125,126]. The knockdown of the myosin 1f (MYOF1) in mast cells downregulated Drp1 activation, reduced mitochondrial fission and mitochondrial translocation to exocytosis sites, and led to a decrease of mast cell degranulation after IgE and MRGPRX2 activation [119].

Furthermore, activation of Drp1 depends on its phosphorylation on Ser616 (at least partly mediated by ERK1/2 activity) and its dephosphorylation on Ser637 by Ca^2+^-dependent phosphatase calcineurin which induces its recruitment to the mitochondrial surface [113]. Treatment with antioxidant SkQ1 decreased IgE-induced mast cell degranulation by inhibiting ERK1/2-dependent mitochondrial fragmentation [109].

Interestingly, this mitochondrial translocation seems to be reversible because mitochondria return in the perinuclear region 24 h after non-IgE stimulation of mast cells [76].

Moreover, vesicles from mast cells contain organelles including mitochondria [117]. IgE and non-IgE mast cell stimulation in vitro and stimulation in rats in vivo led to rapid mast cell degranulation and extracellular small mitochondrial particles secretion near the mast cell surface, which should act as autocrine triggers of degranulation and paracrine triggers to cytokine release [114].

In summary, taken together, these results indicate that IgE and non-IgE mast cell activation leads to mitochondrial fragmentation and to a transient translocation of mitochondria to the mast cell surface near granule exocytosis sites, linked to Drp1 activation (Figure 6). Besides, antigen-stimulated mast cells seem to operate mitochondrial morphological changes with highly folded and shrunken inner membrane cristae.

## 6. Targeting Mitochondria and Oxidative Stress in Anaphylaxis

Mitochondria-targeted therapies showed promise in several settings, including in pulmonary hypertension and septic shock [127,128], showing similarities with anaphylaxis situations. However, mitochondria-targeted therapy to treat anaphylaxis remains poorly investigated.

Several treatments with antioxidant properties have been explored to reduce mast cell degranulation and allergic response. A whey-based diet, which significantly enhanced antioxidant glutathione (GSH) levels in the liver of pigs, reduced allergen-induced anaphylactic bronchoconstriction in sensitized animals by 45% as compared with control-diet pig groups [88]. Besides, although GSH peroxidase mimetic agent ebselen has been shown to inhibit IgE-mediated ROS production in a dose-dependent manner, it did not reduce histamine and β-hexosaminidase release following IgE stimulation [98]. In the same way, synthetic antioxidant Didox treatment decreased oxidative stress, enhanced antioxidant enzymes such as catalase and SOD1 expression, and suppressed pro-inflammatory cytokine production and secretion (IL6, IL13, TNF, MIPa) in mast cells after allergen stimulation [106]. These results were confirmed in a mouse model in vivo by reduction of temperature drop during anaphylaxis when mice were treated with Didox before allergen challenge [106]. The antioxidant SkQ1 has also shown to be effective in reducing degranulation from mast cells after in vitro stimulation and in reducing histamine release from tissue mast cells in an experimental mouse subcutaneous air-pouch model [107]. However, it does not seem to change ROS production induced by allergen stimulation in mast cells [109]. The polyphenol rosmarinic acid, shown to have potential anti-allergic activity through inhibiting IgE-induced ROS production and mast cell degranulation, was confirmed in a PCA mouse model [111]. The antioxidants tetramethylthiourea and N-acetylcysteine (NAC) reduced ROS generation and degranulation induced by non-IgE mast cell stimulation [99]. However, NAC treatment did not significantly reduce mast cell degranulation following IgE stimulation [104].

Besides in vitro results, few in vivo therapeutic approaches are actually available. We reported results based on recent studies of our team. In the setting of anaphylactic shock, the addition of mitochondrial-targeted therapy to current treatment strategies could benefit by restoring the mitochondrial respiration function and the cardiac function [86]. When NButGT was administered in a pretreatment strategy and in association with epinephrine and vascular filling to treat AS, we observed a restoration in OXPHOS through complexes I and II, and an improvement in cardiac function (LV contractility and cardiac output).

Targeting mitochondria and oxidative stress in anaphylaxis may deliver a number of advantages. Indeed, in the setting of cardiac dysfunction, it is well established that treatments mimicking mitochondrial substrates (adenosine receptor agonists, mitochondrial-targeting natural compounds) or mitochondrial antioxidants (mitoTEMPO, mitoQ) could benefit the patient by restoring the normal metabolic milieu and reverting the decline in cardiac substrate usage capabilities [129]. As anaphylaxis and especially AS are characterized by a massive cardiac dysfunction, targeting mitochondria and oxidative stress seems to be a novel therapeutic approach.

## 7. Conclusions and Future Perspectives

The pathophysiology of anaphylaxis implies a complex association of various signaling pathways, and oxidative stress and mitochondria are involved at many stages of AS and mast cell degranulation. AS was linked to oxidative damage and mitochondrial respiration impairment. ROS production from OXPHOS and NOX induced by mast cell stimulation induced their degranulation. Further, mitochondrial respiration, fragmentation, and translocation near the cell surface were key regulators of mast cell degranulation and accordingly showed morphological changes during anaphylaxis.

Some limitations of this systematic review could relate to the focus on mast cell degranulation concerning cellular studies. However, mast cells are the most explored cell lines in this context.

Although mast cell degranulation mechanisms are widely explored, further investigations should prove interesting to expand knowledge about mitochondria and oxidative stress involvement during anaphylaxis. Indeed, few studies investigated mitochondria or oxidative stress during in vivo anaphylaxis, and even fewer of them have assessed it in humans. Since antioxidants were efficient to treat allergic-driven atopic dermatitis [130,131], modulating both oxidative stress and mitochondria should be promising as new therapeutic avenues.

## Figures and Tables

**Table 1:** Experimental studies in animal models and their relevant findings concerning hemodynamic, oxidative stress, oxidative damage and mitochondrial respiration involvement during anaphylaxis. Studies are presented in chronological order. AS: anaphylactic shock, CO: cardiac output, GSH: glutathione, IgG: immunoglobulin G, IgE: immunoglobulin E, MAP: mean arterial pressure, NA: not available, OVA: ovalbumin, OXPHOS: oxidative phosphorylation, SOD: superoxide dismutase, TBARs: thiobarbituric acid reactive substances.

Models	Hemodynamic Signs	Oxidative Stress Antioxidant System	Oxidative Damage	Mitochondria	Publications
IgE-mediated Guinea pigs cardiomyocytes	Tachycardia at first, then bradycardia with conduction disorders	NA	NA	Shrunken or disrupted mitochondria with close cristae and dense matrix	Suzuki et al., 1972 [92]
48/80-induced AS Rats Hepatocytes	NA	NA	NA	Significant decrease in mitochondrial granules	Dimlich et al., 1985 [93]
IgG-mediated Guinea pigs Lung	Anaphylactic airway contraction	Increased or decreased lung GSH level is associated with higher or lower airway hyperreactivity	Increase in lipid peroxidation in pulmonary tract (TBARs)	NA	Kloek et al., 2011 [88]
IgE-mediated Guinea pigs Isolated heart	NA	Increase in superoxide anion radical O2^•−^ and in H_2_O_2_	NA	NA	Rosic et al., 2014 [91]
IgE-mediated Mice Isolated heart	Coronary flow decrease	Cardiac decrease in NO production and no change in O2^•−^ within 2 min after OVA challenge	Cardiac lipid peroxidation (TBARs) decrease within 2 min after OVA challenge	NA	Milicic et al., 2016 [90]
IgE-mediated Mice Hepatocytes	Body temperature decrease	Increase in H_2_O_2_ production Aconitase inactivation Increase in SOD activity	NA	OXPHOS impairment through complexes I and II	Trinchese et al., 2018 [89]
IgE-mediated Brown Norway rats Cardiomyocytes	Severe and rapid decrease in MAP	Trend towards increase in peroxynitrite Increase in SOD activity	Increase in lipid peroxidation (TBARs)	Mitochondrial respiration impairment through complexes I and II	Oulehri et al., 2022 [5]
IgE-mediated Wistar rats Plasma	Severe decrease in MAP	Increase in SOD plasmatic level No difference in catalase activity	No change in plasmatic lipid peroxidation (TBARs) level	NA	Bellou et al., 2022 [87]
IgE-mediated Brown Norway rats Cardiomyocytes	Severe and rapid decrease in MAP, CO and cardiac contractility	NA	NA	Early (15 min) OXPHOS impairment through complex II Delayed (60 min) impairment through complexes I and II	Oulehri et al., 2024 [86]

**Table 2:** Studies investigating mast cell degranulation in relation with oxidative stress and lipid peroxidation. Studies are listed in chronological order. BMMCs, Bone marrow–derived mast cells; [Ca^2+^]_IC_, intracellular calcium; CD63, cluster of differentiation 63 (membrane protein); EGCG, epigallocatechin-3-O-gallate; FSMCs, fetal skin-derived mast cells; H_2_O_2_ hydrogen peroxide; HL, human leucocytes; HCMCs, human peripheral blood–derived cultured mast cells; IgE immunoglobulin E; IC, intracellular; LAD2, laboratory of allergic diseases 2; MFI, mean fluorescence intensity; MPMCs, mouse peritoneal mast cells; mtROS, mitochondrial ROS; NA, not available; NADPH, Nicotinamide adenine dinucleotide phosphate; NAC,N-Acetylcysteine; NIH-3T3, fibroblast cell line; PHK, primary human keratinocytes; PI3K, phosphatidylinositol 3 kinase; PLCγ, phospholipase Cγ; PKC, protein kinase C; RBL-2H3, rat basophilic leukemia; ROS, reactive oxygen species; ROS_IC_, intracellular ROS; SkQ1, cationic plastoquinone derivative 1 (antioxidant effect); SOD 1, superoxide dismutase copper–zinc [Cu–Zn]; TCS, triclosan; UCP2, uncoupling protein.

Cells	Mast Cells Activation	Assessment of Mast Cell Degranulation	Oxidative Stress	Lipid Peroxidation	Publications
RBL-2H3 cells, HL	IgE	histamine release	ROS production in stimulated cells	NA	Yoshimaru T et al., 2002 [97]
RBL-2H3 cells, mice BMMCs	IgE	β-hexosaminidase release	ROS production in stimulated mast cells is mediated by PI3K and NADPH activity; PLCγ is ROS dependent	NA	Suzuki Y et al., 2003 [98]
RBL-2H3 cells	non-IgE	β-hexosaminidase, serotonin and histamine release	Production of ROS during degranulation; tetramethylthiourea and NAC blocked ROS generation and degranulation	NA	Collaco CR et al., 2006 [99]
RBL-2H3 cells	IgE	β-hexosaminidase release	Production of ROS during degranulation	NA	Yasui Y et al., 2007 [100]
mice BMMCs, RBL-2H3 cells	IgE and non-IgE	β-hexosaminidase release	IgE stimulation increased ROS production via NADPH oxidase, but to a lesser extent following non-IgE activation	NA	Inoue, T., et al., 2008 [101]
RBL-2H3 cells	IgE and non-IgE	β-hexosaminidase and histamine release, β-hexosaminidase activity	complex I and II inhibitors decreased mtROS generation after stimulation; complex III inhibitor increased H_2_O_2_ secretion; NAC decreased spontaneous and mtROS-induced degranulation; decrease in mtROS or ROS_IC_ levels reduced degranulation; mtROS regulated PKC activity	NA	Chodaczek G et al., 2009 [79]
RBL-2H3 cells	IgE	β-hexosaminidase release	Hydrogen attenuated NADPH oxidase activity, decreased antigen-induced production of H_2_O_2_ and O^2−^	NA	Itoh et al., 2009 [102]
BMMCs, FSMCs, LAD2 cells	IgE and non-IgE	histamine release	UCP2 knockdown enhanced ROS production, SOD-mimetic treatment reduced degranulation	NA	Tagen M et al., 2009 [103]
RBL-2H3 cells, mice BMMCs	IgE	β-hexosaminidase release	EGCG enhanced antigen-induced ROS production	EGCG induced cardiolipin oxidation	Inoue T et al., 2010 [80]
mice BMMCs, HCMCs	IgE	β-hexosaminidase release	Increase in [ROS]_IC_ levels after stimulation; ROS_IC_ production depends on [Ca^2+^]_IC_	NA	Zhou Y et al., 2013 [104]
RBL-2H3 cells	IgE and non-IgE	serotonin release	Acrolein increased ROS_IC_ in dose-dependent manner; NAC reduced ROS production and degranulation	NA	Hochman DJ et al., 2014 [105]
mice BMMCs, MPMCs	IgE	CD63 MFI and cytokine release	Didox decreased oxidative stress, enhanced catalase and SOD1 expression	NA	McLeod JJA et al., 2017 [106]
MPMCs, RBL-2H3 cells	IgE and non-IgE	β-hexosaminidase activity	SkQ1 inhibited degranulation	NA	Chelombitko MA et al., 2017 [107]
PHK, NIH-3T3 mouse fibroblast, RBL-2H3 cells	IgE	NA	ROS production in stimulated cells; TCS mildly increased ROS production of stimulated cells	NA	Weatherly LM et al., 2018 [84]
RBL-2H3 cells	IgE	β-hexosaminidase release	PM2.5 increased ROS levels and degranulation	NA	Wang Y et al., 2021 [108]
RBL 2H3 cells	IgE	β-hexosaminidase release	SkQ1 decreased degranulation but did not change ROS production	NA	Pavlyuchenkova AN et al., 2022 [109]
RBL-2H3 cells	IgE	β-hexosaminidase release	Mitocur 1 and 3 increased ROS levels	Mitocur 1 and 3 increased cardiolipin peroxidation	Pavlyuchenkova AN et al., 2023 [110]
RBL-2H3 cells	IgE	β-hexosaminidase and histamine release	Rosmarinic downregulated ROS overproduction during degranulation	NA	Jia B et al., 2024 [111]

**Table 3:** In vitro studies investigating mast cell degranulation in relation with mitochondrial respiration, mitochondrial transcription factors and mtDNA, calcium flux, and mitochondrial dynamic and morphology. Studies are listed in chronological order. AD, Atopic dermatitis; AMA, Antimycin A; ATP, adenosine triphosphate; BMMCs, bone marrow–derived mast cells; CC, cetylpyridinium chloride; Ca^2+^, calcium ion; [Ca^2+^]IC, intracellular calcium; [Ca^2+^]m, mitochondrial calcium; CD63, cluster of differentiation 63 (membrane protein); CRAC, calcium release-activated channels; Drp1, dynamin-related protein 1; EGCG, epigallocatechin-3-O-gallate; FCCP, carbonyl cyanide-4-(trifluoromethoxy)phenylhydrazone; FSMCs, fetal skin-derived mast cells; hCBMCs, human cord blood–derived mast cells; HL, human leucocytes; HCMCs, human peripheral blood–derived cultured mast cells; HMC, human mast cells; hSKM, human skin mast cells; IC, intracellular; IgE, immunoglobulin E; LAD2, laboratory of allergic diseases 2; MCU, mitochondrial calcium uniporter; MFI, mean fluorescence intensity; MITF, microphthalmia-associated transcription factor; mtDNA, mitochondrial DNA; MPMCs, mouse peritoneal mast cells; MRGPRX2, Mas-related G-protein coupled receptor member X2; MYOF1, myosin 1f; NA, not available; NIH-3T3, fibroblast cell line; OCR, oxygen consumption rate; OMY, oligomycin; PDH, pyruvate dehydrogenase; PHK, primary human keratinocytes; PI3K, phosphatidylinositol 3 kinase; RBL-2H3, rat basophilic leukemia; ROS, reactive oxygen species; ROT, rotenone; STAT3, signal transducer and activator of transcription 3; SkQ1, cationic plastoquinone derivative 1 (antioxidant effect); TCS, triclosan; TNF, tumor necrosis factor; UCP2, uncoupling protein 2.

Cells	Mast Cells Activation	Assessment of Mast Cell Degranulation	Mitochondrial Respiration	Mitochondrial Transcription Factors and mtDNA	Calcium Flux	Mitochondrial Dynamic and Morphology	Publications
RBL-2H3 cells, mice BMMCs	IgE	β-hexosaminidase release	ROT had no inhibitory effect on IgE-induced ROS production	NA	inhibition of IgE-mediated ROS production reduced Ca^2+^ influx	NA	Suzuki Y et al., 2003 [98]
RBL-2H3 cells, mice BMMCs	IgE and non-IgE	β-hexosaminidase release	ROT, AMA, OMY and FCCP decreased IgE degranulation; ROT and FCCP reduced non-IgE degranulation	NA	IgE stimulation induced [Ca^2+^]_m_ drop	NA	Suzuki et al., 2006 [75]
RBL-2H3 cells, mice BMMCs	IgE and non-IgE	NA	NA	NA	increase in [Ca^2+^]_m_ in activated cells; mitochondria take up Ca^2+^ via MCU	NA	Suzuki Y, 2008 [112]
mice BMMCs, RBL-2H3 cells	IgE and non-IgE	β-hexosaminidase release	NA	NA	ROS production depends on [Ca^2+^]_m_	NA	Inoue, T., et al., 2008 [101]
RBL-2H3 cells	IgE and non-IgE	β-hexosaminidase and histamine release, β-hexosaminidase activity	complex I and II inhibitors inhibited degranulation; complex III inhibitor enhanced degranulation	NA	NA	NA	Chodaczek G et al., 2009 [79]
BMMCs, FSMCs, LAD2 cells	IgE and non-IgE	histamine release	UCP2 knockdown enhanced degranulation	NA	NA	NA	Tagen M et al., 2009 [103]
RBL-2H3 cells, mice BMMCs	IgE	β-hexosaminidase release	high concentration FCCP suppressed degranulation	NA	EGCG induced mtCa^2+^ release	NA	Inoue T et al., 2010 [80]
hCBMCs, LAD2 cells, hSKM from control and patients with AD	IgE and non-IgE	β-hexosaminidase release	NA	NA	Drp1 activation depends on [Ca^2+^]_IC_ increase	mitochondrial translocation near cell surface; Drp1 inhibitor inhibits mitochondrial translocation and degranulation	Zhang B et al., 2011 [113]
LAD2 cells, hCBMCs	IgE and non-IgE	β-hexosaminidase release	OMY inhibited TNF secretion	NA	NA	transient mitochondrial translocation near cell surface	Zhang B et al., 2012 [76]
RBL-2H3 cells	IgE	β-hexosaminidase release	ROT and AMA inhibited degranulation	NA	ROT and AMA reduced the [Ca^2+^]_m_ uptake after stimulation	NA	Takekawa M et al., 2012 [78]
LAD2 cells, hCBMCs	IgE and non-IgE	β-hexosaminidase and histamine release	NA	NA	NA	mitochondrial translocation near cell surface	Zhang B et al., 2012 [114]
mice BMMCs, HCMCs	IgE	β-hexosaminidase release	NA	NA	increase in [Ca^2+^]_IC_ in activated cells	NA	Zhou Y et al., 2013 [104]
RBL-2H3 cells, mice BMMCs, hCBMCs	IgE	β-hexosaminidase release	major part of energy for degranulation derived from mitochondrial ATP	STAT3 inhibition abolished degranulation; mtSTAT3 phosphorylation in activated cells	NA	NA	Erlich et al., 2014 [82]
RBL-2H3 cells	IgE	β-hexosaminidase release	NA	NA	MCU needed for mast cell degranulation	NA	Furuno T et al., 2015 [115]
PHK, HMC, NIH-3T3 mice fibroblasts, RBL-2H3 cells	IgE and non-IgE	β-hexosaminidase release	TCS inhibited ATP production and degranulation	NA	NA	NA	Weatherly LM et al., 2016 [83]
mice BMMCs	non-IgE	tryptase release	NA	NA	stimulation increased both [Ca^2+^]_IC_ and [Ca^2+^]_m_; MCU blocker reduced degranulation	NA	Cuong DV et al., 2016 [116]
RBL-2H3 cells	IgE	cryofluorescence and soft X-ray tomography to determine location of granule	NA	NA	NA	translocation of mitochondria to cell surface; morphological changes of mitochondria	Chen HY et al., 2016 [117]
RBL-2H3 cells, BMMCs, hCBMCs	IgE	β-hexosaminidase release	PDH inhibition reduced mitochondrial ATP levels in a dose-dependent manner and degranulation	PDH dephosphorylation and detachment from MITF in activated cells	NA	NA	Sharkia I et al., 2017 [85]
mice BMMCs, MPMCs	IgE	CD63 MFI and cytokine release	Didox suppressed cytokine release and CD63 MFI	NA	NA	NA	McLeod JJA et al., 2017 [106]
PHK, NIH-3T3 mouse fibroblast, RBL-2H3 cells	IgE	NA	TCS decreased ATP production	NA	TCS disrupts mitochondrial Ca^2+^ buffering capacity	TCS inhibits mitochondrial translocation in stimulated cells	Weatherly LM et al., 2018 [84]
RBL-2H3 cells, BMMC, hCBMCs	IgE	β hexosaminidase release and IL-6 release	Mitocur 1 and 3 reduced mitochondrial ATP levels in a dose-dependent manner and reduced OCR	Mitocur 1 and 3 reduced degranulation	NA	NA	Erlich TH et al., 2018 [118]
RBL-2H3 cells, Jurkat T cells	IgE	β hexosaminidase release	NA	NA	TCS inhibited degranulation by inhibiting CRAC channel	NA	Sangroula S et al., 2020 [81]
LAD2 cells	IgE and non-IgE	β-hexosaminidase release	NA	NA	NA	MYOF1 knockdown decreased degranulation	Navinés-Ferrer A et al., 2021 [119]
RBL-2H3, LAD2 cells, MPMCs	IgE and non-IgE	β-hexosaminidase release	NA	NA	Forsythiae fractus decreased [Ca^2+^]_IC_ by enhancing [Ca^2+^]_m_ and reduced degranulation	NA	Qi R et al., 2021 [120]
human mast cells from intestinal tissue	IgE	β-hexosaminidase release	NA	Resveratrol inhibited mtSTAT3 phosphorylation and reduced degranulation	NA	NA	Bilotta S et al., 2021 [121]
mice BMMCs	IgE and non-IgE	β-hexosaminidase release	OMY decreased AgNP-induced degranulation but not IgE- nor C48/80-induced degranulation	NA	NA	NA	Mendoza et al., 2021 [77]
RBL 2H3 cells	IgE	β-hexosaminidase release	NA	NA	NA	SkQ1 inhibited Erk1/2-dependent mitochondrial fragmentation	Pavlyuchenkova AN et al., 2022 [109]
RBL-2H3 cells, mice BMMCs	IgE	β-hexosaminidase release	increased OXPHOS activity in stimulated cells	phosphorylated MITF in activated cells	NA	NA	Paruchuru LB et al., 2022 [122]
hSKM	IgE	β-hexosaminidase release	increased OCR in activated cells; no effect of inhibition of complex 3 on degranulation; inhibition of complex I decreased degranulation	NA	NA	NA	Buttgereit T et al., 2022 [74]
RBL-2H3 cells	IgE	β-hexosaminidase release	NA	Mitocur 1 and 3 reduced STAT3 phosphorylation in activated cells	NA	Mitocur 1 and 3 caused mitochondrial fragmentation and swelling	Pavlyuchenkova AN et al., 2023 [110]
RBL-2H3 cells	non-IgE	β-hexosaminidase release	NA	NA	CC inhibits degranulation by decreasing Ca^2+^ efflux from RE, reduces [Ca^2+^]_m_ and [Ca^2+^]_IC_	NA	Obeng B et al., 2023 [123]
LAD2 cells	non-IgE	β-hexosaminidase release	NA	MRGPRX2 activation increased MITF phosphorylation and activity; MITF silencing reduced degranulation	NA	NA	Guo Y et al., 2023 [124]
RBL-2H3 cells	IgE	β-hexosaminidase and histamine release	NA	Rosmarinic down-regulated mRNA expression of genes implicated in the oxidative stress signaling pathway (NQO1, Nrf2, HO-1) of degranulated cells	Rosmarinic inhibited flux of [Ca^2+^]_IC_ in stimulated mast cells	NA	Jia B et al., 2024 [111]

## Data Availability

Data collection forms and data extracted from included studies are available from the authors upon request.

## Supplementary Materials

The following supporting information can be downloaded at: https://www.mdpi.com/article/10.3390/antiox13080920/s1. Supplementary S1: MEDLINE PubMed Search Strategy.
