# Supplementary material for: Oxidative Stress and Mitochondria Are Involved in Anaphylaxis and Mast Cell Degranulation: A Systematic Review
Source: Antioxidants (Basel). 2024 Jul 29;13(8):920. doi: 10.3390/antiox13080920 (PMC11352116; doi:10.3390/antiox13080920)
Supplement: Supplementary file 1 [file antioxidants-13-00920-s001.zip › antioxidants-3083049-supplementary.pdf]

SUPPLEMENTARY MATERIAL

**Supplementary S1. MEDLINE PubMed Search Strategy**

(‘mitochondria’) AND (‘mast cell degranulation’)
(‘mitochondrial respiration’) AND (‘mast cell degranulation’)
(oxidative stress) AND (‘mast cell degranulation’)
(‘mitochondria’ OR ‘mitochondrial respiration’ OR ‘oxidative stress’) and (‘mast cell degranulation’)
(‘mitochondria’ OR ‘mitochondrial respiration’) AND (‘mast cell degranulation’)
(‘mitochondria’ OR ‘oxidative stress’) and (‘mast cell degranulation’)
(‘mitochondrial respiration’ OR ‘oxidative stress’) AND (‘mast cell degranulation’)
('mitochondria' OR ‘mitochondrial respiration’ OR 'oxidative stress') AND ('anaphylaxis' OR 'anaphylactic shock' OR 'allergic shock')
('mitochondria') AND ('anaphylaxis' OR 'anaphylactic shock' OR 'allergic shock')
('mitochondria' OR 'oxidative stress') AND ('anaphylaxis' OR 'anaphylactic shock' OR 'allergic shock')
('mitochondria' OR ‘mitochondrial respiration’) AND ('anaphylaxis' OR 'anaphylactic shock' OR 'allergic shock')
(‘mitochondrial respiration’) AND ('anaphylaxis' OR 'anaphylactic shock' OR 'allergic shock')
(‘mitochondrial respiration’ OR 'oxidative stress') AND ('anaphylaxis' OR 'anaphylactic shock' OR 'allergic shock')
('oxidative stress') AND ('anaphylaxis' OR 'anaphylactic shock' OR 'allergic shock')
('mitochondria') AND ('anaphylaxis')
('mitochondria') AND (‘anaphylactic shock')
('mitochondria') AND (‘allergic shock')
('mitochondria') AND (‘anaphylaxis' OR 'allergic shock')
('mitochondria') AND (‘anaphylaxis' OR 'anaphylactic shock')
('mitochondria') AND (‘anaphylactic shock' OR 'allergic shock')
('mitochondrial respiration') AND ('anaphylaxis')
('mitochondrial respiration') AND (‘anaphylactic shock')
('mitochondrial respiration') AND (‘allergic shock')
('mitochondrial respiration') AND (‘anaphylaxis' OR 'allergic shock')
('mitochondrial respiration') AND (‘anaphylaxis' OR 'anaphylactic shock')
('mitochondrial respiration') AND (‘anaphylactic shock' OR 'allergic shock')
(‘oxidative stress’) AND ('anaphylaxis')
(‘oxidative stress’) AND (‘anaphylactic shock')
(‘oxidative stress’) AND (‘allergic shock')
(‘oxidative stress’) AND (‘anaphylaxis' OR 'allergic shock')
(‘oxidative stress’) AND (‘anaphylaxis' OR 'anaphylactic shock')
(‘oxidative stress’) AND (‘anaphylactic shock' OR 'allergic shock')
